# Post‐term births as a risk factor for small for gestational age births and infant mortality in Brazil, Mexico, and Palestinian refugees: An analysis of electronic birth records

**DOI:** 10.1111/ppe.13137

**Published:** 2024-11-17

**Authors:** Zeina Jamaluddine, Lorena Suarez Idueta, Enny S. Paixao, Julia M. Pescarini, Hala Ghattas, Miho Sato, Akihiro Seita, Luis A. Martinez‐Juarez, Mauricio L. Barreto, Eric O. Ohuma, Louise T. Day, Oona M. R. Campbell, Hannah Blencowe

**Affiliations:** ^1^ Faculty of Epidemiology and Population Health London School of Hygiene & Tropical Medicine London UK; ^2^ School of Tropical Medicine and Global Health Nagasaki University Nagasaki Japan; ^3^ Mexican Society of Public Health Mexico City Mexico; ^4^ Centre for Data and Knowledge Integration for Health (CIDACS) Fundação Oswaldo Cruz Salvador Brazil; ^5^ Department of Health Promotion, Education, and Behavior University of South Carolina Columbia South Carolina USA; ^6^ United Nations Relief and Works Agency for Palestinian Refugees in the Near East Amman Jordan

**Keywords:** electronic records, foetal growth restriction, induction, infant mortality, middle‐income countries, post‐term gestation, small for gestational age

## Abstract

**Background:**

Post‐term pregnancy, defined as reaching or exceeding 42 + 0 weeks of gestation, is known to be associated with unfavourable birth outcomes. High‐income countries have responded to this risk by widely adopting labour induction protocols in late‐term, but many low‐ and middle‐income countries have not. However, understanding underlying mechanisms linking post‐term births to adverse newborn and infant outcomes remains limited.

**Objective:**

To investigate the (a) prevalence of post‐term, (b) the risk factors associated with post‐term (c) the association between post‐term births and the risk of small‐for‐gestational‐age (SGA) neonates and of infant mortality in middle‐income settings.

**Methods:**

We used existing electronic datasets from the general population of Brazil, Mexico, and Palestinian refugees. Regression models were used to explore the associations between post‐term birth and SGA and infant mortality.

**Results:**

We analysed 21,335,033 live births in Brazil (2011–2018), 23,416,126 in Mexico (2008–2019), and 966,102 in Palestinian refugees (2010–2020) (*N* = 45,717,261). Post‐term deliveries accounted for 3.1% of births in Brazil, 1.2% in Mexico, and 2.1% in Palestinian refugees. Post‐term births had approximately three times the risk of resulting in SGA neonates compared to term births. Additionally, post‐term neonates exhibited a 15% to 40% increased risk of infant mortality compared to term infants. Notably, post‐term SGA neonates faced a significantly increased risk of infant mortality compared to term appropriate for gestational age neonates.

**Conclusions:**

These findings emphasise the critical significance of implementing induction strategies to prevent post‐term pregnancies and mitigate the associated risks of SGA neonates and subsequent infant mortality. Moreover, the study highlights the importance of accurately determining gestational age and using INTERGROWTH‐21st charts to improve the identification of SGA cases, enabling targeted interventions. This is especially relevant because post‐term SGA neonates may not exhibit low birthweight (a commonly used risk marker) and, therefore, may miss out on required specialised attention.

**Synopsis**

**Study Question**

What is the prevalence of post‐term pregnancy? What is the association between post‐term pregnancies and the risk of small‐for‐gestational‐age (SGA) neonates and of infant mortality?

**What is already known**

Post‐term pregnancies are associated with adverse birth outcomes. Preterm is the greatest contributor to infant mortality, however, more research is needed to estimate the contribution of SGA to infant mortality when pregnancies reach 42 weeks of gestation or beyond. Mechanisms linking post‐term to adverse newborn and infant outcomes are poorly understood. High‐income nations have adopted labour induction protocols and thus have very few post‐term births. In many middle and low‐income countries, pregnancies commonly extend beyond term.

**What the study adds**

This study highlights the persistent issue of post‐term births even in settings able to estimate gestational age. It indicates that women with less education are more likely to have post‐term birth and that post‐term birth increases the risk of SGA and infant mortality. It emphasises the need for proactive induction strategies before pregnancies reach the post‐term stage. It also underscores the importance of accurately measuring gestational age and supports the use of international growth standards, e.g., INTERGROWTH‐21st charts, to identify those with SGA and at increased risk of infant mortality for appropriate interventions.

## 1 BACKGROUND

Post‐term pregnancy, defined as gestation of 42 + 0 weeks or more, is associated with adverse birth outcomes.^1^ Post‐term pregnancy can lead to placental insufficiency, which compromises the oxygen and nutrient supply, resulting in foetal growth restriction; this in turn can lead to stillbirth, small size for gestational age at birth, and subsequent mortality for newborns. Whilst post‐term pregnancies are relatively uncommon, they carry a significant risk of preventable mortality. Guidelines aimed at reducing stillbirths recommend inducing labour at 41 + 0 weeks gestation,^2^, ^3^ or as early as 39 + 0 weeks.^4^, ^5^, ^6^ These practices are widely adopted in higher‐income countries, resulting in few post‐term births.

In low‐ and middle‐income countries (LMIC), where the highest burden of adverse perinatal and infant outcomes lies, little is known about the prevalence of post‐term births, its risk factors, or its consequences. In part, this is because some LMIC countries do not record gestational age well (using low birthweight (<2500 g) as a proxy for neonatal risk instead) and thus have little information on post‐term birth. However, some middle‐income countries are recording gestational age on a routine basis.

It is increasingly recognised that combining gestational age and birthweight to identify babies which are small for gestational age (SGA) is a more accurate predictor of early morbidity and mortality than using a birthweight threshold.^7^ Additionally, global standards for size for gestational age were only made available recently via the development of global foetal and newborn size charts based on a large, diverse, and representative sample of pregnancies from different populations by the International Fetal and Newborn Growth Consortium for the 21st Century (INTERGROWTH‐21st). This has led to suitable benchmarks from LMICs and facilitated international comparisons.^8^, ^9^

A recent study spanning 23 countries generated six newborn types, focusing on preterm versus term and three sizes at birth (SGA, appropriate for gestational age [AGA], and large for gestational age [LGA]).^7^, ^10^ They found SGA babies had an increased mortality risk compared to AGA in both preterm and term gestations^10^ (excluding post‐term). They did not investigate the relationship for post‐term neonates.

In this study, we used data from Brazil, Mexico, and Palestinian refugees in Jordan, Lebanon, Syria, the West Bank, and Gaza to expand the six newborn types to nine by including post‐term SGA, AGA and LGA dimensions. These populations record gestational age and birthweight and reflect contexts where there are no national policies in place to promote induction before post‐term is reached.

We estimated the prevalence of post‐term, and whether (1) maternal education and age increased the risk of post‐term, (2) whether post‐term pregnancy increased the risk of SGA and (3) whether post‐term or post‐term SGA increased the risk of infant mortality.

## 2 METHODS

### 2.1 Data sources

We analysed routinely collected individual‐level electronic data of births in Brazil, Mexico, and Palestine refugees. Brazilian and Mexican data are national, while Palestinian refugees' data are from Jordan, Lebanon, Syria, West Bank and Gaza. Palestinian refugees are protracted refugees (hosted in these five settings since 1948) and they are best characterised as a largely urban poor population. Information on GDP per capita and infant mortality rate in each of the countries, showing broad commonality, is presented in Supporting Information.

#### 2.1.1 Brazilian data

We extracted data from 1 January 2011 to 31 December 2018 from Brazil's Live Birth Information System (Sistema de Informa*ções* sobre Nascidos Vivos—SINASC) obtained from the Centre for Data and Knowledge Integration for Health (CIDACS). SINASC is a nationwide registry, based on the Declaration of Birth, a mandatory document filled by the birth assistant, i.e., a healthcare professional. SINASC covers over 95% of all live births in Brazil and includes information on mothers (age, parity, educational attainment, place of residence, marital status, race/skin colour, obstetric history) and live birth (sex, birthweight, length of gestation, multiples and presence of congenital anomalies).^11^

#### 2.1.2 Mexican data

We extracted data from 1 January 2008 to 31 December 2019 from Mexico's National Information Subsystem of Livebirths (SINAC), a public dataset administered by the Ministry of Health. We included all live births and deaths registered. SINAC includes information on mothers (age, parity, educational attainment, antenatal care), live births (sex, birthweight, length of gestational age, multiple), and health care facilities (type of facility and state/municipality of delivery), with an estimated coverage of 90% from the target population.^12^ Data on foetal and neonatal deaths were extracted from the National Institute of Statistics and Geography (INEGI). Livebirth and death certificates are mandatory in the country and collected by healthcare workers or administrative personnel.^13^ To assess infant mortality, we used a previously linked dataset with live births and deaths from day 0 to 365 after birth.^14^

#### 2.1.3 Palestinian refugee data

The United Nations Relief and Works Agency for Palestinian refugees in the Near East (UNWRA) provides free primary healthcare services to Palestinian refugees in Jordan, Lebanon, Syria, West Bank, and Gaza, including free antenatal care services. The Palestinian refugee population is not composed of recent refugees but rather represents a more stable, urban poor population. We extracted anonymised obstetric record data from UNRWA electronic medical records between 1 January 2010, and 31 December 2020.^15^ Obstetric records of pregnancy outcomes were collected retrospectively during postnatal care, the child's first vaccination visit, and by telephone follow‐up and are included in the electronic medical records. Detailed pregnancy outcomes included birthweight, gestational age at delivery, mode of delivery, malpresentation, multiplicity (twins etc) and sex of the child.

In all three datasets, gestational age was estimated from the last menstrual period (LMP) or ultrasound. However, the estimation method was not recorded in the data, nor was there a no record of accuracy (e.g., the trimester of the first ultrasound or the certainty of the LMP).

### 2.2 Inclusion and exclusion

All pregnancies, including singletons and multiples, resulting in at least one live birth, were included in the main analysis. Stillbirths were excluded as comparable data were not available in Brazil.

The data quality of birthweight and gestational age measurement were explored in detail in previous studies for Brazil, Mexico, and Palestinian refugees,^10^, ^16^ including assessments of data, heaping, digit preference, and range of values. We excluded values with extreme/implausible birthweights (more than 6 kg) or gestational ages (less than 22 weeks and more than 44 weeks).

### 2.3 Newborn types

We defined nine newborn types based on three newborn gestational categories: preterm birth (between 22 + 0 and 36 + 6 weeks), term birth (37 + 0 to 41 + 6 weeks), and post‐term (42 + 0 to 44 + 6 weeks) and size for gestational age. We assigned size at birth using birthweight, gestational age, and sex according to the INTERGROWTH‐21st standards into SGA, <10th percentile, AGA, 10th to 90th percentiles, and LGA, >90th percentile.^8^, ^9^, ^10^, ^17^ The nine resulting newborn types were: preterm‐SGA, preterm‐AGA, preterm‐LGA, term‐SGA, term‐AGA, term‐LGA, post‐term‐SGA, post‐term‐AGA, and post‐term‐LGA.

### 2.4 Statistical analysis

We assessed the prevalence of post‐term and of the nine newborn types for Brazil, Mexico, and Palestinian refugees. We used log‐binomial regression models to estimate relative risks (RR) for the association between maternal characteristics (education and age at delivery) and post‐term birth, excluding preterm pregnancies. Log‐binomial regression models were employed to explore the association between post‐term birth and the outcomes of SGA or AGA, excluding LGA. We used generalised linear models with log‐binomial regression to estimate the relative risk for the association between the nine newborn types and infant mortality (death in the first year of life). Special focus was given to understanding risks for post‐term and post‐term SGA infants. We adjusted the Palestinian model for location, including Gaza, Jordan, Lebanon, Syria, and the West Bank.

Finally, we calculated the population attributable risk (PAR) for each exposure with the following formula where *pr* is the prevalence and *RR* is the relative risk:

$$\begin{array}{c} \mathrm{PAR}\;\text{for type of interest}=\frac{\mathrm{pr}\left(\text{type of interest}\right)\left(\mathrm{RR}\left(\text{type of interest}-1\right)\right)}{\sum_{\mathrm{all}\;\text{types}}\mathrm{pr}\left(\text{type}\right)\times \mathrm{RR}\left(\text{type}\right)} \end{array}$$

All analyses were adjusted for the study setting and were undertaken using Stata (StataCorp, College Station, TX, USA) and R statistical software.

### 2.5 Missing data and analysis datasets

**FIGURE 1:**
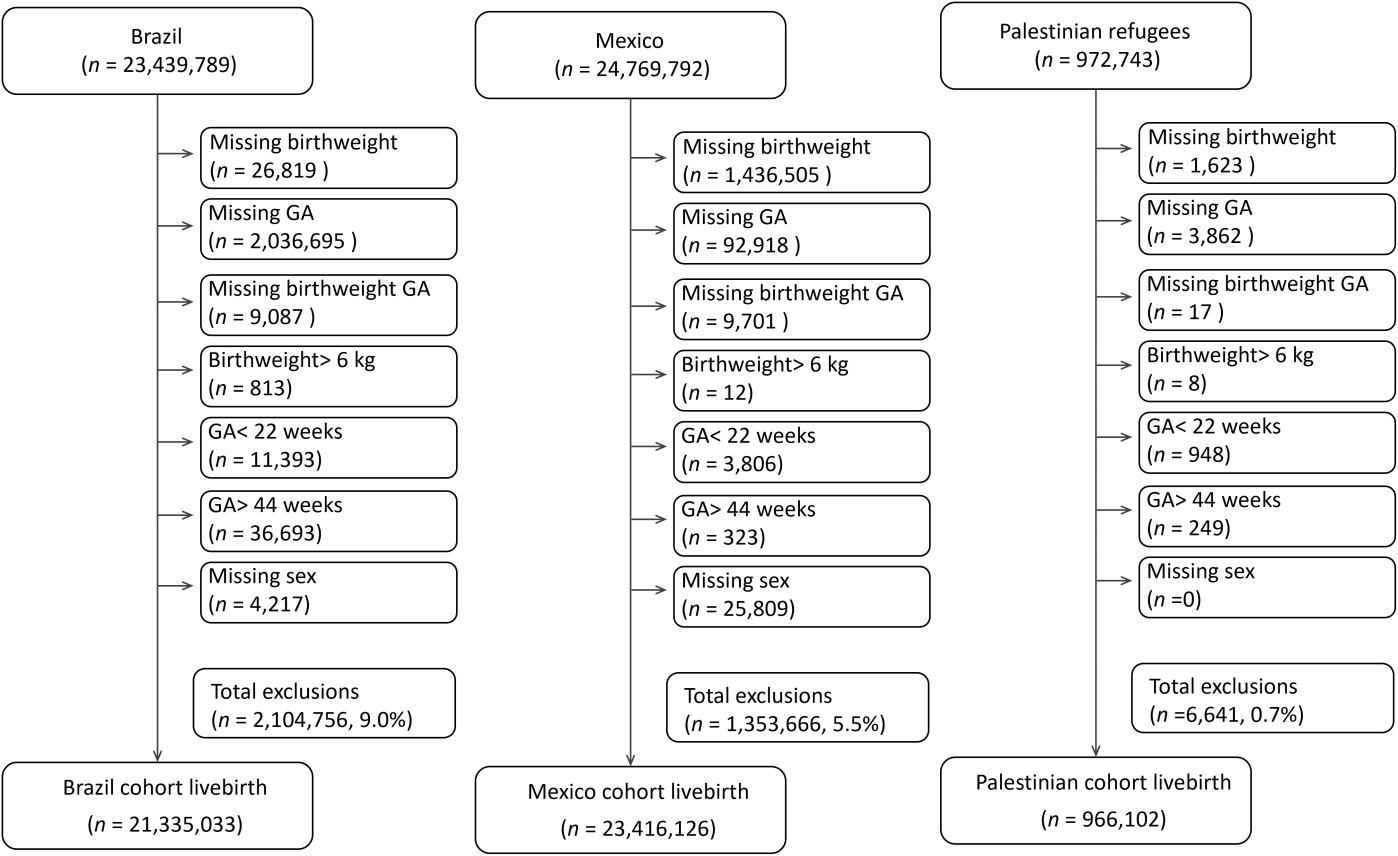
Flow chart describing total birth and exclusion in the three datasets (some cases in the exclusion might have overlaps).

The percentage of missing data was limited: 9.0% for the Brazil dataset, 5.5% for Mexico, and 0.7% for Palestinian refugees (Figure 1). We excluded these missing entries. When exploring the association of maternal education and age with post‐term births, the comparator was term birth. When investigating the association between post‐term births and SGA, the comparator was AGA. The exclusion of preterm births and LGA births respectively decreased the sample size.

### 2.6 Sensitivity analysis

We conducted a sensitivity analysis of the association between post‐term and SGA using a sub‐analysis considering SGA <3rd percentile, LGA >97th percentile and excluding multiple pregnancies.

For the Palestinian refugee dataset, as records were extracted in August 2021, the youngest children (born after September 2020) had a truncated time period for the risk of infant mortality. We conducted a sensitivity analysis by only including those with a full year of follow‐up.

### 2.7 Ethics approval

Ethical approval was obtained from the Federal University of Bahia's Institute of Public Health Ethics Committee (reference number 18022319.4.0000.5030), the Centre of Investigation in Health Sciences, Anahuac University, Mexico (reference number 202214), UNRWA Research Board and London School of Hygiene and Tropical Medicine (reference number 25467). We used administrative de‐identified data to analyse the data, consent was waived by ethical boards.

## 3 RESULTS

The three datasets included a total of 49,373,861 births among which 23,439,789 were in Brazil, 24,955,172 in Mexico, and 978,900 in the five settings hosting Palestinian refugees. The Brazil data did not include stillbirths, so we excluded them in the Mexican and Palestinian datasets. The final dataset for analysis was 45,717,261 live births (93.0% of the dataset live births) after excluding data with missing gestational age or birthweight, extreme birthweights or gestational ages and missing sex (Figure 1).

Among the three studied populations, Brazil showed the highest prevalence of post‐term births, followed by Palestinian refugees, with Mexico having the lowest prevalence (Table 1). For SGA births, Palestinian refugees had the highest prevalence, followed closely by Brazil, with Mexico showing the lowest prevalence (Table 1). The pattern for post‐term SGA births mirrored that of overall post‐term births, with Brazil having the highest prevalence, followed by Palestinian refugees, and Mexico with the lowest (Table 1). In all three datasets, women with higher education levels (secondary, diploma, or university level education) had lower risks of post‐term live birth compared to those with lower educational levels (Table 2). Maternal age was not associated with post‐term livebirth in the Mexican or Palestinian datasets. In Brazil, older women had a lower risk of post‐term livebirth as compared to women aged less than 20 (Table 2).

**TABLE 1:** Prevalence of the nine types of newborns, among livebirths in three datasets.

	Brazil		Mexico		Palestinian refugees	
	*(N* = 21,335,033)	%	*(N* = 23,416,126)	%	*(N* = 966,102)	%
Gestational age						
Preterm	2,465,209	11.6	1,499,502	6.4	78,467	8.2
Post‐term	669,704	3.1	268,651	1.2	20,458	2.1
Size for gestational age						
SGA	1,699,048	8.0	1,661,891	7.1	89,682	9.3
LGA	3,464,621	16.2	2,145,224	9.2	102,875	10.7
Nine types of size for gestational age						
Preterm						
Preterm‐SGA	182,696	0.9	143,423	0.6	7477	0.8
Preterm‐AGA	1,491,029	7.0	1,246,947	5.3	53,986	5.6
Preterm‐LGA	791,484	3.7	109,132	0.5	17,004	1.8
Term						
Term‐SGA	1,338,224	6.3	1,453,089	6.2	76,772	8.0
Term‐AGA	14,22,424	67.4	18,174,206	77.6	705,857	73.0
Term‐LGA	2,639,472	12.5	2,020,678	8.6	84,548	8.8
Post‐term						
Post‐term‐SGA	178,128	0.8	65,379	0.3	5433	0.6
Post‐term‐AGA	457,911	2.2	187,858	0.8	13,702	1.4
Post‐term‐LGA	33,665	0.2	15,414	0.1	1323	0.1

Abbreviations: AGA, appropriate for gestational age; LGA, large for gestational age; SGA, small for gestational age.

**TABLE 2:** Maternal age and education level: prevalence and association with post‐term pregnancy (excluding preterm) in all three datasets.

	Brazil		Mexico		Palestinian refugees^a^	
	Prevalence excluding preterm (%)	Relative risk for being post‐term (excluding preterm) RR (95% CI)	Prevalence excluding preterm (%)	Relative risk for being post‐term (excluding preterm) RR (95% CI)	Prevalence excluding preterm (%) All sample	Relative risk for being post‐term (excluding preterm) RR (95% CI)
Maternal education level^b^	*N* = 18,613,172	*N* = 18,613,172	*N* = 21,597,782	*N* = 21,597,782	*N* = 847,953	*N* = 847,953
Basic	22.6	1.00 (Reference)	14.5	1.00 (Reference)	32.3	1.00 (Reference)
Secondary	59.1	0.64 (0.63, 0.64)	56.8	0.81 (0.80, 0.82)	42.7	0.84 (0.82, 0.87)
Bachelor's and above	18.3	0.28 (0.28, 0.29)	27.1	0.51 (0.50, 0.52)	25.0	0.71 (0.68, 0.74)
Maternal age	*N* = 18,884,790	*N* = 18,884,790	*N* = 21,856,780	*N* = 21,856,780	*N* = 887,506	*N* = 887,506
Under 20 years old	17.5	1.00 (Reference)	19.9	1.00 (Reference)	9.8	1.00 (Reference)
20–24 years	25.5	0.89 (0.89, 0.90)	29.8	1.01 (1.00, 1.02)	25.9	1.01 (0.96, 1.07)
25–29 years	24.5	0.73(0.73, 0.74)	24.6	0.95 (0.94, 0.96)	31.8	1.05 (1.00, 1.10)
30–34 years	19.8	0.57 (0.56, 0.57)	16.2	0.92 (0.90, 0.93)	19.7	1.03 (0.98, 1.09)
35–39 years	10.2	0.48 (0.48, 0.49)	7.5	0.91 (0.90, 0.93)	9.8	1.01 (0.95, 1.07)
40–44 years	2.3	0.50 (0.49, 0.51)	1.6	0.99 (0.96, 1.02)	2.8	0.96 (0.88, 1.06)
45 plus	0.2	0.62 (0.58, 0.66)	0.1	1.10 (0.99, 1.20)	0.3	0.61 (0.43, 0.85)

Abbreviations: CI, confidence interval; RR, relative risk.

^a^ Adjusted for setting where Palestinian refugee reside.

^b^ Maternal education level was defined by context as in Brazil (0, 7, 8, 12, 12+ years), Mexico (primary and lower secondary (≤11 years), upper secondary and academy professional degree (12, 14 years) and bachelor's and above (≥15 years)) and Palestinian refugees (basic, then intermediate secondary diploma then university and higher degrees.).

**FIGURE 2:**
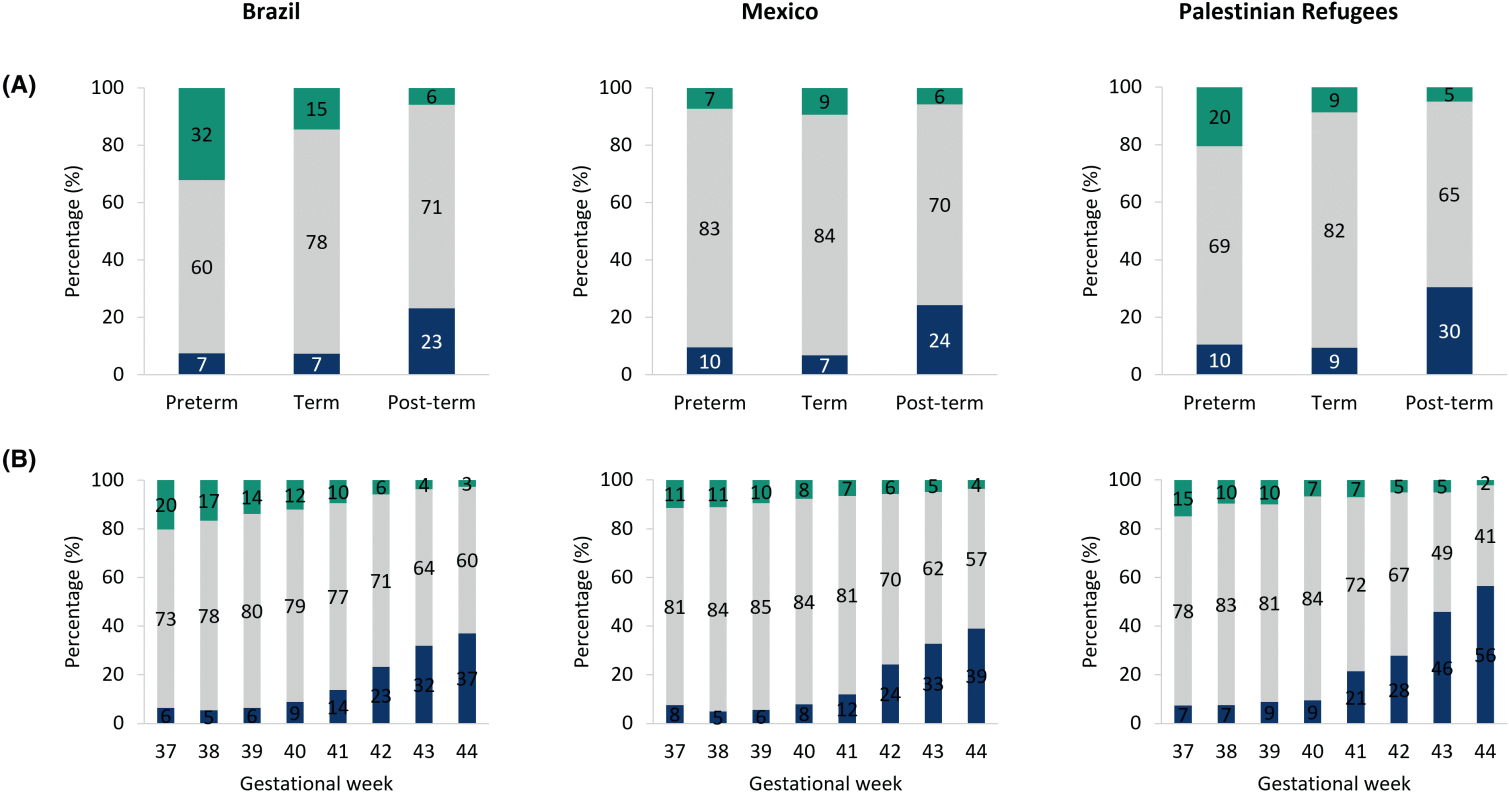
Distribution of size for gestational age by (A) preterm, term and post‐term live births and (B) by week of gestation for term and post‐term live births in Brazil, Mexico, and Palestinian refugees. (blue SGA, grey AGA, green LGA)

Post‐term live births had around three times the risk of being SGA compared to those born at term (Table 3). Analyses of size for gestational age categories by week of gestation showed an increased prevalence of SGA births from 41 weeks onwards in all three datasets (Figure 2). Post‐term infants had an increased risk of infant mortality compared to term in Brazil (Table 4). In all three datasets, post‐term SGA newborns in particular had higher risks of infant mortality as compared to term AGA in Brazil, Mexico and Palestinian refugees (Table 4). Sensitivity analyses excluding infants with less than 1 year follow‐up showed similar results to the main analyses (Supporting Information). An analysis focusing on infants classified SGA below the 3rd percentile and LGA above the 97th percentile showed similar results (Supporting Information) as did sub‐analyses excluding multiple pregnancies (Supporting Information). At the population level, most infant deaths were attributed to preterm‐AGA, whilst a smaller portion of infant mortality was attributed to post‐term births, or post‐term SGA (Table 4).

**TABLE 3:** Association of post‐term with small for gestational (SGA) (excluding large for gestational age) in all three datasets.

	Brazil	Mexico	Palestinian refugees^a^
	Relative risk (95% CI)	Relative risk (95% CI)	Relative risk (95% CI)
	*N* = 17,870,412	*N* = 21,270,902	*N* = 863,227
Preterm	1.27 (1.26, 1.28)	1.40 (1.39, 1.40)	1.23 (1.20, 1.25)
Term	1.00 (Reference)	1.00 (Reference)	1.00 (Reference)
Post‐term	3.25 (3.24, 3.27)	3.49 (3.46, 4.51)	3.04 (2.97, 3.11)

Abbreviation: CI, confidence interval.

^a^ Adjusted for setting where Palestinian refugees reside.

**TABLE 4:** Relative risk of the association between nine newborn types and infant mortality in all three datasets (SGA is highlighted in blue) and population attributable factor (PAR).

	Brazil		Mexico		Palestinian refugees^a^	
	Relative risk (95% CI)	PAR % (95% CI)	Relative risk (95% CI)	PAR % (95% CI)	Relative risk (95% CI)	PAR % (95% CI)
	*(N* = 21,308,005)		*(N* = 23,416,126)		(*N* = 966,102)	
Gestational age						
Preterm	12.11 (12.00, 12.21)	56.0 (55.8, 56.2)	3.21 (3.17, 3.25)	12.4 (12.2, 12.6)	9.76 (9.43, 10.11)	41.7 (40.9, 42.6)
Ter (Reference)	1.00 (Reference)		1.00 (Reference)		1.00 (Reference)	
Post‐term	1.39 (1.34, 1.43)	0.7 (0.6, 0.8)	1.16 (1.12, 1.22)	0.2 (0.1, 0.2)	1.15 (0.99, 1.33)	0.2 (0.0, 0.4)
Size at birth						
SGA	3.61 (3.57, 3.64)	17.6 (17.4, 17.7)	1.25 (1.23, 1.27)	1.8 (1.6, 1.9)	2.72 (2.60, 2.84)	12.5 (12.0, 13.2)
AGA (Reference)	1.00 (Reference)		1.00 (Reference)		1.00 (Reference)	
LGA	0.87 (0.86, 0.88)	−1.7 (−1.8, −1.5)	0.91 (0.90, 0.93)	−0.6 (−0.7, −0.5)	2.08 (1.9, 2.19)	9.1 (7.7, 9.8)
Nine newborn types						
Preterm‐SGA	42.08(41.50, 42.67)	13.3 (13.2, 13.3)	3.78 (3.66, 3.91)	1.4 (1.4, 1.5)	20.94 (19.64, 22.33)	7.9 (7.6, 8.1)
Preterm‐AGA	15.28 (15.11, 15.45)	35.9 (35.8, 35.9)	3.26 (3.22, 3.30)	10.4 (10.2, 10.5)	9.31 (8.90, 9.73)	23.0 (22.7, 23.2)
Preterm‐LGA	6.01 (5.98, 6.11)	6.7 (6.7, 6.7)	2.34 (2.24, 2.46)	0.6 (0.5, 0.6)	14.13 (13.36, 14.93)	11.7 (11.4, 11.9)
Term‐SGA	4.48 (4.41, 4.55)	7.9 (7.8, 7.9)	1.20 (1.18, 1.22)	1.1 (1.0, 1.2)	2.88 (2.71, 3.06)	7.4 (7.0, 7.8)
Term‐AGA (Reference)	1.00 (Reference)		1.00 (Reference)		1.00 (Reference)	
Term‐LGA	0.85 (0.83, 0.87)	−0.7 (−0.8, −0.6)	0.97 (0.96, 0.99)	−0.2 (−0.3, −0.1)	1.05 (0.96, 1.14)	0.2 (−0.2, 0.6)
Post‐term‐SGA	3.15 (3.01, 3.29)	0.6 (0.6, 0.7)	1.34 (1.24, 1.46)	0.1 (0.1, 0.1)	2.09 (1.67, 2.63)	0.3 (0.2, 0.5)
Post‐term‐AGA	1.16 (1.11, 1.22)	0.1 (0.1, 0.2)	1.14 (1.08, 1.20)	0.1 (0.1, 0.1)	1.06 (0.86, 1.30)	0.0 (−0.1, 0.2)
Post‐term‐LGA	1.55 (1.34, 1.79)	0.0 (0.0, 0.1)	0.96 (0.79, 1.17)	0.0 (0.0, 0.0)	1.08 (0.56, 2.07)	0.0 (0.0, 0.1)

Abbreviations: AGA, appropriate for gestational age; CI, confidence interval; LGA, large for gestational age; PAR, population attributable factor; SGA, small for gestational age.

^a^ Adjusted for settings where Palestinian refugees reside.

## 4 COMMENT

### 4.1 Principal findings

This study explores post‐term births and the risk of SGA and infant mortality in three middle‐income country datasets. We found the prevalences of post‐term were between 1.2% and 3.1%, and the prevalence of post‐term SGA was between 0.3% and 0.8%. Maternal education increased the risk of post‐term birth and post‐term birth was associated with a three‐fold increase in the risk of SGA. We also found that being born post‐term increased the risk of infant mortality compared to term, and post‐term SGA was associated with an almost doubling of the risk of death in infancy compared to term AGA. These findings have important implications for policies to reduce post‐term births to reduce preventable mortality and morbidity.

### 4.2 Strengths of the study

The availability of large electronic datasets enabled us to investigate the associations between post‐term and post‐term‐SGA and the adverse outcomes of SGA and mortality in three datasets, even though post‐term and post‐term‐SGA pregnancies are relatively uncommon.

### 4.3 Limitations of the data

We recognise the limitations of our analysis, many due to well‐known challenges of collecting and using routine birth data. Gestational age may have been inaccurate, as the datasets contained a combination of the LMP and ultrasound measurements without distinguishing between them or reporting the gestational age at ultrasound dating. Repeating this analysis including only those with more certain gestational age assessment, e.g., based on first‐trimester ultrasound or certain LMP would have been useful, but was not possible.^18^ Nevertheless, while measurement error might explain the higher prevalence of SGA, it is unlikely to explain the mortality associations seen for post‐term SGA newborns compared to term‐ AGA.

Birthweight, which should be measured with standard processes (within 1 h of birth, calibrated scales etc) is sub‐optimally measured in many LMIC settings, and heaping of birthweight heaping may have contributed to over‐ and under‐estimates of SGA newborns.^19^

In addition, errors might result from using the INTERGROWTH 21st chart for gestational ages above 43 weeks which are based on extrapolation. However, we observed a notable increase in SGA even at 41 and 42 gestational weeks included in the original INTERGROWTH‐21st charts.

While the largest effect of post‐term is on stillbirth risk, stillbirth data is not available for Brazil, and we excluded it for comparative analysis across the settings.

### 4.4 Interpretation

The prevalence of post‐term in our populations is at the lower end of global estimates, possibly because of the high rates of caesarean sections in these settings.^20^ Although the prevalence of post‐term pregnancies is relatively low, the associated risks are high. The 3.1% proportion of post‐term births observed in Brazil corresponds to 669,709 live births, a significant number. This translates to a preventable mortality of 0.5% PAR in infant mortality, suggesting that addressing these cases can lead to better health outcomes. The exact causes of post‐term birth remain unclear, but some studies have suggested that hormonal and genetic factors and obesity may play a role.^21^, ^22^, ^23^ Irrespective of the biological causes, access to good quality care following guidelines should avert post‐term births. In our study, we showed that higher maternal education is associated with a reduced risk of post‐term birth, suggesting that socioeconomic factors enable access to medical care. In this analysis, women with higher education levels were more likely to receive quality antenatal care, and a higher number of visits with first‐trimester gestational age assessment, potentially expediting their delivery post‐date.^24^, ^25^, ^26^

While social disadvantage is an important factor in overall health outcomes (particularly in post‐term pregnancies), it is not the primary driver of the increased risk of infant mortality associated with post‐term pregnancies.

As pregnancy progresses beyond the expected delivery date, there is a higher likelihood of placental dysfunction because of infarction, fibrin deposition, and calcification.^27^ In some cases, placental function might further deteriorate if exacerbated by infections or by chronic mild hypertension.^27^ Placental dysfunction compromises the placental ability to efficiently transfer oxygen and essential nutrients to the developing foetus.^27^ Consequently, the foetus experiences inadequate nourishment and may result in foetal growth restriction manifested as SGA, this is seen in Mexico, Brazil, and Palestinian refugees. This may also explain the higher stillbirth rates seen in post‐term pregnancies reported in the literature.^1^

Our study also indicated that post‐term and post‐term SGA newborns have an elevated risk of infant mortality in the first year of life compared to infants born at term and at AGA term respectively. The increased risk of mortality in post‐term SGA infants may be attributed to various complications, including birth asphyxia, respiratory distress syndrome (e.g., meconium aspiration syndrome), increased susceptibility to infections and hypoglycaemia cause mortality.^1^, ^28^ In addition, post‐term and LGA are associated with birth trauma, increased hospitalisation, cerebral palsy, Wilms tumour and others.^29^

The effect size from the three populations indicates a consistent pattern of association, however, differences in the exact magnitude of the effects between the three datasets may be due to unmeasured and thus uncontrolled confounding factors, such as maternal obesity, which are associated with the exposure of being post‐term and the outcome of neonatal mortality^30^ and which may differ in prevalence among the three populations. In addition, potential misclassification due to methods used for gestational age measurement may have impacted comparability. Notably, Mexico shows the greatest divergence, likely due to challenges with gestational age measurement in this setting.

In terms of implications, the study highlights the need for (1) increased access to antenatal care to better assess gestational age and potentially identify SGA in utero, (2) induction to avert post‐term births (3) and postnatal assessment of size for gestational age, to ensure that SGA babies with birthweights <2500 grams are recognised as being at higher risk and managed appropriately.

Our data indicate that there is a need to increase the induction of labour before post‐term in middle‐income countries. The results of this study are generalizable to other contexts where pregnancies reach post‐term stage. This requires accurately measuring gestational age, to prevent inadvertent iatrogenic preterm or early‐term birth associated with inaccurate assessments. Another clinical implication is the need to adopt internationally comparable growth charts e.g., INTERGROWTH21^st^ newborn standards are a useful tool to assist in the effective identification of SGA, and hence increased risk including postnatally and especially in post‐term babies. These standards are increasingly being adopted and we utilised them in our analyses to facilitate international comparisons.^7^

Recent guidelines by the World Health Organisation (WHO) and The National Institute for Health and Care Excellence (NICE) advocate for induction at 41 weeks of gestation,^2^, ^3^ and show the potential to reduce perinatal complications without increasing caesarean section rates. It is particularly important it is possible to do this without increasing caesarean prevalence, among Brazil, Mexico, and Palestinian refugees, as rates are already high.^16^, ^31^, ^32^, ^33^ Implementing a policy of inducing at 41 weeks in Brazil, Mexico, and the settings where Palestinian refugees reside could contribute to reducing preventable mortality and morbidity. In settings where a national policy for induction at 41 weeks is not well established, and there are plans to introduce one, ensuring proper access to first‐trimester antenatal care and accurate gestational age assessment using LMP and ultrasound is essential to ensure women are not induced too soon. Challenges in implementing ultrasound measurements in middle‐income countries must be addressed to optimise perinatal care.^34^

Postnatal care for post‐term SGA newborns may be overlooked when relying solely on birthweight for risk stratification, as happens with current newborn risk stratification in many low‐ and middle‐income countries. Using low birthweight <2500 g, misses post‐term SGA babies as they are unlikely to be low birthweight and more likely to be normal or even high birthweight. For example, a 2850 g female newborn or a 3000 g male newborn born at 42 weeks of gestation (post‐term) would be classified as having a normal birth weight but is actually at the 5th percentile on the INTERGROWTH21 charts and is classified as SGA. For example, in the Palestinian data, focusing on low birthweight alone would miss 6% of the SGA births that are not low birthweight; among post‐term births, the comparable figure is 25% that are SGA but not low birthweight. Our study demonstrates the value of the birthweight for gestational age percentile charts over low birthweight thresholds in helping to identify post‐term SGA newborns, and so enabling tailored neonatal care and interventions (e.g., regular blood glucose monitoring) for this vulnerable subgroup.^35^

Future research should focus on understanding the causes and timing of infant mortality among post‐term SGA newborns. Despite the primary focus of our study on post‐term SGA, we recognise the significance of exploring the implications of LGA prevalence on adverse outcomes, particularly in countries experiencing nutritional transitions and rising obesity rates. Finally, understanding the association between post‐term SGA and future risk factors, including chronic diseases, remains a relatively unexplored area in the existing literature.^29^

## 5 CONCLUSIONS

In conclusion, this study analyses routine data from middle‐income settings and extends the previously used vulnerable newborn types to highlight, post‐term births and their, association with poorer outcomes of SGA and infant mortality at 1 year.

These findings emphasise the necessity for policy strategies targeting the reduction of post‐term pregnancies. This will require scaling up of universal first‐trimester gestational age measurement, developing and implementing labour induction strategies for pregnancies at 41 weeks gestation and assessing size at birth for all to enable appropriate risk assessment and identify additional care needs.

## AUTHOR CONTRIBUTIONS

ZJ and OC conceived the study. ZJ, LI, EP and JP cleaned the data. ZJ, LI and EP, analysed the data for Palestinian refugees (ZJ), Mexico (LI) and Brazil (EP), with guidance from OC and HB. ZJ, HB and OC wrote the first draft of the manuscript. EO provided the extrapolation of the INTERGROWTH21. LTD, HG, MS, AS, LM and MB supported in interpreting the findings and writing the analysis. All authors read and approved the final version.

## FUNDING INFORMATION

Zeina Jamaluddine was supported by the Nagasaki University “Doctoral Program for World‐leading Innovative and Smart Education” for Global Health, KYOIKU KENKYU SHIEN KEIHI, Ministry of Education, Culture, Sports, Science and Technology (MEXT). Enny Paixao is funded by the Wellcome Trust. The funder had no role in study design, data collection, data analysis, data interpretation, or writing.

## CONFLICT OF INTEREST STATEMENT

The authors report no conflict of interest.

## Supporting information


Data S1.

## Data Availability

The data that support the findings of this study are available on request from the corresponding author. The data are not publicly available due to privacy or ethical restrictions.
