# Supplementary material for: Post‐term births as a risk factor for small for gestational age births and infant mortality in Brazil, Mexico, and Palestinian refugees: An analysis of electronic birth records
Source: Paediatr Perinat Epidemiol. 2024 Nov 17;39(2):149–58. doi: 10.1111/ppe.13137 (PMC11866736; doi:10.1111/ppe.13137)
Supplement: Supplementary file 1 — Data S1. [file PPE-39-149-s001.docx]

**Supplementary material**

Supplementary material Table 1- GDP per capita and infant mortality rate in each of the six countries

| National level data | Brazil | Mexico | Jordan | Lebanon | Syria | Palestine (West Bank and Gaza) |
| --- | --- | --- | --- | --- | --- | --- |
| GDP per capita (37) | 9,670 USD in 2023 | 12,670 USD in 2023 | 5,050 USD in 2023 | 3,590 USD in 2020 | 2,810 USD in 2010 | 3,510 USD in 2023 |
| Infant mortality rate  Death per 1,000 livebirths (38). | 12.9 in 2021 | 11.4 in 2021 | 12.6 in 2021 | 7.1 in 2021 | 18.4 in 2021 | 12.8 in 2021 |

Supplementary material Figure 1. Distribution of size for gestational age (with SGA <3^rd^ percentile and LGA>97^th^ percentile) by preterm, term and post-term live births

**
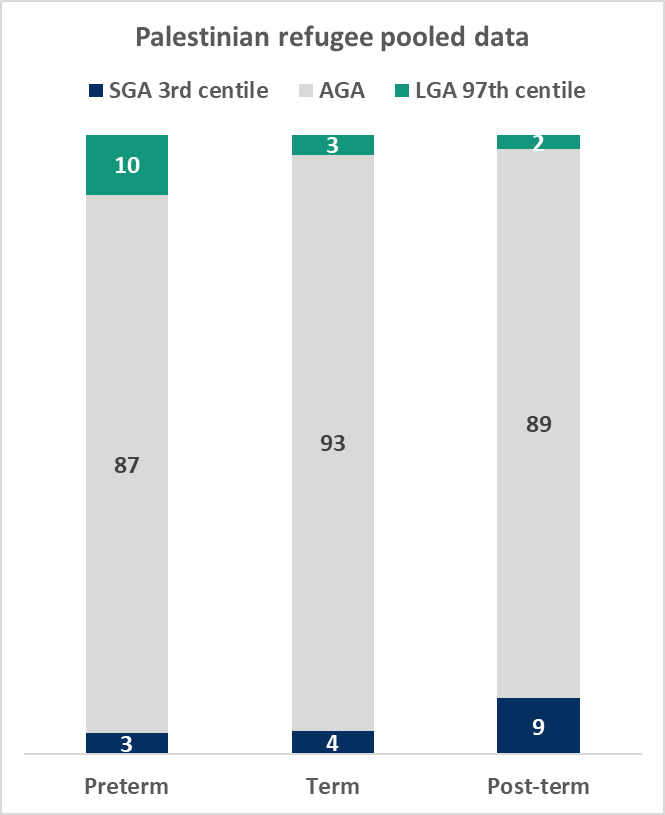
**

Supplementary material Table 2. Association of post-term with small for gestational (SGA) among singleton.

|  | **Palestinian refugees** relative risk ratio (95% CI) |
| --- | --- |
|  | N= 845,700 |
| Preterm | 1.11 (1.08,1.14) |
| Term | 1.00 (Reference) |
| Post-term | 3.14 (3.06,3.21) |

^a^adjusted for setting where Palestinian refugees reside.

Supplementary material Table 3. Relative risk ratio of the association between nine newborn types and infant mortality (SGA is highlighted in blue) among singleton.

|  | **Palestinian refugees ^a^** |
| --- | --- |
|  | Relative risk (95% CI) |
|  | N= 947,823 |
| Gestational age |  |
| Preterm | 9.96 (9.61,10.33) |
| Term  (Reference) | 1.00 (Reference) |
| Post-term | 1.16 (1.00,1.35) |
| Size at birth |  |
| SGA | 2.76 (2.64,2.80) |
| AGA (Reference) | 1.00 (Reference) |
| LGA | 2.07 (1.96,2.17) |
| Nine newborn types |  |
| Preterm-SGA | 23.98 (22,41,25.67) |
| Preterm-AGA | 9.47 9.05,9.91) |
| Preterm-LGA | 13.73 (12.97,14.53) |
| Term-SGA | 2.90 (2.73,3.08) |
| Term-AGA (Reference) | 1.00 (Reference) |
| Term-LGA | 1.05 (0.96,1.14) |
| Post-term-SGA | 2.11 (1.67,2.63) |
| Post-term-AGA | 1.06 (0.86,1.30) |
| Post-term-LGA | 1.08 (0.56,2.08) |

Supplementary material Table 5. Relative risk ratio of the association between nine newborn types and infant mortality (SGA is highlighted in blue) (Palestinian refugee cohort 2010-2019)

|  | **Palestinian refugees ^a^** |
| --- | --- |
|  | Relative risk ratio (95% CI) |
|  | N=881,018 |
| Gestational age |  |
| Preterm | 9.86 (9.51, 10.23) |
| Term  (Reference) | 1.00 (Reference) |
| Post-term | 1.14 (0.98, 1.33) |
| Size at birth |  |
| SGA | 2.67 (2.55,2.80) |
| AGA (Reference) | 1.00 (Reference) |
| LGA | 2.15(2.05,2.26) |
| Nine newborn types |  |
| Preterm-SGA | 20.39 (19.03,21.83) |
| Preterm-AGA | 9.42 (8.99,9.86) |
| Preterm-LGA | 14.32 (13.51,15.17) |
| Term-SGA | 2.84 (2.67,3.02) |
| Term-AGA (Reference) | 1.00 (Reference) |
| Term-LGA | 1.07 (0.98,1.18) |
| Post-term-SGA | 2.04 (1.61,2.60) |
| Post-term-AGA | 1.06 (0.86,1.32) |
| Post-term-LGA | 1.15 (0.60,2.20) |

^a^ adjusted for settings where Palestinian refugees reside.
